# Relationship between Sperm Parameters with Sperm Function Tests in Infertile Men with At Least One Failed Intracytoplasmic Sperm Injection Cycle

**DOI:** 10.22074/ijfs.2020.5750

**Published:** 2019-11-11

**Authors:** Farzaneh Bassiri, Mohammad Hossein Nasr-Esfahani, Mohsen Forozanfar, Marziyeh Tavalaee

**Affiliations:** 1 Department of Biology, Fars Science and Research Branch, Islamic Azad University, Fars, Iran; 2 Department of Biology, Shiraz Branch, Islamic Azad University, Shiraz, Iran; 3 Department of Reproductive Biotechnology, Reproductive Biomedicine Research Center, Royan Institute for Biotechnology, ACECR, Isfahan, Iran; 4 Isfahan Fertility and Infertility Center, Isfahan, Iran

**Keywords:** DNA Damage, Intracytoplasmic Sperm Injections Spermatozoa, Lipid Peroxidation, Protamines

## Abstract

**Background:**

Imbalance between production of reactive oxygen species (ROS) and total antioxidant capacity in testis, epididymis, and seminal fluid can eventually lead to infertility. Abnormal sperm chromatin packaging, and DNA fragmentation is considered as the main underlying causes of infertility. Therefore, we aimed to assess relationship between sperm parameters with DNA damage, protamine deficiency, persistent histones, and lipid peroxidation in infertile men with at least one failed cycle after intracytoplasmic sperm injection (ICSI).

**Materials and Methods:**

In this experimental study, semen samples were collected from infertile men with at least one failed intracytoplasmic sperm injection (ICSI) cycle (n=20). Sperm parameters, DNA damage, protamine de- ficiency, persistent histones, and lipid peroxidation were assessed using computer-assisted sperm analysis (CASA) system, sperm chromatin structure assay (SCSA) and Terminal deoxynucleotidyl transferase dUTP nick end labeling (TUNEL) assays, chromomycin A3, aniline blue, and BODIPY C11 staining, respectively.

**Results:**

A negative significant correlation was observed between sperm concentration with percentage of sperm persistent histones (r=-0.56, P=0.02), while positive significant correlations were found between percentage of sperm persistent histones with percentage of abnormal morphology (r=0.54, P=0.02), CMA3-positive spermatozoa (r=o.6, P=0.008) and intensity of lipid peroxidation (r=0.6, P=0.01). In addition, significant correlation was observed be- tween sperm DNA damage with intensity and percentage of lipid peroxidation (r=0.62, P=0.009). Correlation between CMA3-positive spermatozoa and intensity of lipid peroxidation (r=0.5, P=0.03) were also significant.

**Conclusion:**

Observed significant correlations between sperm functional tests in infertile men with at least one failed ICSI cycle, indicated that the reduction of oxidative stress by antioxidant supplementation may be considered as one therapy approach that can improve sperm function and increase the chance of successful clinical outcomes in next assisted reproductive cycle.

## Introduction

One of the main byproducts of sperm metabolism is reactive oxygen species (ROS). Distinct roles have been envisaged for ROS at physiological and pathological levels. According to literature, a basal level of ROS are needed for processes such as sperm capacitation, acrosome reaction and sperm-oocyte fusion. But, uncontrolled or excess production of ROS can have devastating effects on sperm functions. Several studies have demonstrated that induction of lipid peroxidation cascades and fragmentation of DNA are two main pathological consequences of ROS production in sperm (1, 2). 

Lipid peroxidation could lead to formation of electrophilic lipid aldehydes such as malondialdehyde, acrolein and 4-hydroxynonenal (4HNE). These aldehydes further increase ROS level through binding to nucleophilic centers of proteins, such as succinic acid dehydrogenase in the mitochondrial electron transport chain (1, 2) and thereby, induce a vicious cycle in production of ROS. The consequence of excessive ROS production is oxidation-induced apoptosis which is dose- and timedependent (3, 4). In this regard, Aitken (5) has recently proposed a two-steps hypothesis which accounts for how DNA fragmentation occurs in sperm. The first step is a defect in the chromatin remodeling taking place during differentiation of spermatid to spermatozoa. This defect lead to relaxation of chromatin compared to when the chromatin is tightly packed. The second step is free radicals attack to the relaxed chromatin configuration. In addition, he stated “free radical attack might occur at any time during the life of a spermatozoon from its differentiation during spermiogenesis to its maturation and storage in the epididymis”. Collectively, these three fundamental aspects may account for etiology of DNA fragmentation in sperm (5-7). In this regard, several studies showed that there are significant correlations between sperm DNA fragmentation (SDF) with low quality of embryo, failed pregnancy and reduced implantation rate in infertile men candidate for assisted reproduction techniques (7). Considering importance of three aforementioned intrinsic factors in relation to DNA damage in sperm, we assessed sperm lipid peroxidation as an important class of generated biomolecules by oxidative stress, DNA fragmentation as a indicator of apoptotic sperm, and protamine deficiency as a chromatin maturity marker in infertile men with at least one failed cycle after ICSI.

## Materials and Methods

### Patients

This experimental study was performed from April 2016 to April 2018, and approved by the Ethics Committee of Royan Institute (IR.ACECR.ROYAN.REC.1396.270). Couples were informed of the study design and all the participants signed a written consent. Semen samples were obtained from 20 individuals referred to Isfahan Fertility and Infertility Center (IFIC) with male factor infertility. 

### Inclusion and exclusion criteria

Inclusion criteria: Couples with male factor infertility and at least one previous failed cycle after ICSI, without sign of varicocele and/reported genetic defect.

Exclusion criteria: Infertile couples with female factor infertility, individuals with leukospermia or varicocele, urinary infection, klinefelter syndrome, cancer and excessive alcohol or drug abuse.

### Semen collection and analysis

Semen samples was collected from 20 infertile men with previous failed cycles after ICSI by masturbation after 3-7 days of abstinence. Part of the semen sample was used for assessment of sperm parameters (concentration, motility, morphology) with computerassisted sperm analysis (CASA) system (Video Test, Version Sperm 2.1©, Russia) according to World Health Organization (WHO) criteria (8). The remaining portion was used for assessment of lipid peroxidation (BODIPY C11 staining), persistent histones (Aniline blue staining), protamine deficiency (CMA3 staining) and DNA fragmentation (SCSA and TUNEL assays).

### Assessment of sperm lipid peroxidation

The level of lipid peroxidation in sperm was evaluated by BODIPY C11 loading BODIPY 581/591 C11 (D3861, Molecular Probes) according to Aitken et al. (9). Briefly, the sperm concentration was adjusted to 2×10^6^ /ml. Equal volume of diluted sperm was mixed with equal volume of BODIPY C11 to have a final concentration of 5 mM. The mixture was maintained at 37°C for 30 minutes. Then, each sperm was washed twice at 650 g for 5 minutes. For positive control, oxidative stress was induced by hydrogen peroxide (H_2_O_2_, 100 µM) after the addition of H_2_O_2_ to sperm suspensions for each sample. Percentage of lipid peroxidation in sperm and intensity of lipid peroxidation in BODIPY C11 positive spermatozoa population were assessed using a FACSCalibur flow cytometer (Becton Dickinson, San Jose, CA, USA). Intensity expresses the average of color emission intensity in cell population in the fluorescence channel. If the lipid peroxidation in the cell is further expressed, the intensity of the color (BODIPY-C11 dye) will increase.

### Assessment of sperm persistent histones

Percentage of persistent histones in sperm samples was assessed by aniline blue staining according to Nasr-Esfahani et al. (10) protocol. Semen samples were washed twice with phosphate-buffered saline (PBS, Merck, Germany) at 300 g for 5 minutes, and two smears for each sample were prepared and air-dried at room temperature. Afterwards, slides were fixed in a solution of 3% glutaraldehyde in 0.2 M phosphate buffer (14 ml of 0.2 M NaH_2_PO_4_ plus 36 ml of 0.2 M Na_2_HPO_4_, pH=7.2) for 30 minutes. Then, slides were stained with solution of 5% aniline blue in 4% acetic acid (pH=3.5) for 5 minutes. Lastly, stained smears were placed in alcohol 50, 70 and 100% for 30 seconds, respectively. For each sample, a minimum of 200 sperm cells were counted. Spermatozoa with unstained nucleus were considered as normal persistence of histones while spermatozoa with dark blue nuclei were considered as abnormal with retention of persistence of histones.

### Assessment of sperm protamine deficiency

Percentage of protamine deficiency was assessed by chromomycin A3 (CMA3) staining according to Razavi et al. (11). Briefly, washed samples with PBS was fixated by Carnoy’s solution (methanol1:3glacial acetic acid) and incubated at 4°C for 5 minutes. Afterward, the sperm suspension was smeared on the slides. Then, prepared smears were treated for 20 minutes with CMA3 solution (McIlvaine buffer), and washed using PBS. Microscopic analysis of the slides was performed by an Olympus fluorescent microscope (Japan) with appropriate filters (460-470 nm). About 200 sperm cells were assessed and sperm with bright yellow color was considered as CMA3- positive spermatozoa with deficient protamine.

### Assessment of sperm DNA fragmentation

Sperm DNA fragmentation was assessed by two procedures; sperm chromatin structure assay (SCSA) and Terminal deoxynucleotidyl transferase dUTP nick end labeling (TUNEL) according to Evenson (12), and Kheirollahi-Kouhestani et al. (13) with minor alternation.

For TUNEL assay: washed semen samples were fixed by 4% paraformaldehyde for 25 minutes and treated with 0.2% Triton X-100 for 5 minutes. Then, samples were washed with PBS and stained with a detection kit of DNA fragmentation (Apoptosis Detection System Fluorescein; Promega, Mannheim, Germany) according to the manufacturer’s instructions. For each sample, one positive control with an additional step [treatment of sperm with DNase I (1,000 U) after permeabilization with 0.2% Triton X-100] was considered for each sample. Finally, a minimum of 10,000 sperm were analyzed using BD Cell Quest Pro software, and the result was reported as TUNEL-positive spermatozoa for each sample. 

For SCSA assay: sperm concentration was adjusted to 2×10^6^ in 1ml of TNE [Tris HCl (Merck, Germany)/NaCl (Merck, Germany)/EDTA (Merck, Germany)] buffer. For test group, 400 μl acid-detergent solution was added to 200 μl of diluted sample in TNE buffere and after 30 seconds, 1200 μl of acridine orange staining solution was mixed with this suspension, while for control group, only 1200 μl of acridine orange (Sigma, St. Louis, USA) staining solution was added to 200 μl of diluted sample. Finally, a minimum of 10,000 sperm for each sample were counted using a FACSCalibur flowcytometer, and the percentage of DNA damage was reported as SCF (14).

### Statistical method

For statistical analysis, correlation coefficients were carried out with the Statistical Package for the Social Sciences software (SPSS 16, SPSS, Chicago, IL, USA). The mean, standard error, and range of variables were presented according to descriptive analysis. P<0.05 was considered significant.

## Results

Description of sperm parameters, and couples age were presented in Table 1. Mean of female and male age were 32.5 ± 6.4 and 37 ± 6.2, respectively. Mean of sperm concentration, percentage of sperm total motility, and abnormal morphology were 46.1 ± 5.4, 34.5 ± 5.09, and 98.1 ± 0.4 respectively.

**Table 1:** Description of sperm parameters, semen volume, and couples age (n=20)


Parameters	Minimum	Maximum	Mean ± SE

Female age (Y)	20	49	32.5 ± 6.1
Male age (Y)	29	51	37 ± 6.1
Sperm concentration (10^6^/ml)	8	80	46.1 ± 5.4
Sperm total motility (%)	5.2	72	34.5 ± 5.09
Sperm abnormal morphology (%)	95	100	98.1 ± 0.4
Semen volume (ml)	2	5.5	4.1 ± 0.2


The correlations analysis between sperm parameters with sperm functional tests such as DNA fragmentation, protamine deficiency, persistent histones, and lipid peroxidation (Table 2) show that there is a negative significant correlation between sperm concentration with percentage of sperm persistent histones (r=- 0.56, P=0.02), while positive significant correlations were observed between percentage of sperm abnormal morphology with percentage of sperm persistent histones (r= 0.54, P=0.02) and intensity of lipid peroxidation (r=0.62, P=0.01). Other correlations were not significant at P<0.05 level.

In addition, correlations between sperm functional tests were analyzed together and results are presented in Table 3. There were significant correlations between percentage of persistent histones with percentage of CMA3- positive spermatozoa (r=o.6, P=0.008) and intensity of sperm lipid peroxidation (r=0.6, P=0.01). We also observed significant positive correlations between percentage of DNA fragmentation assessed by SCSA (DFI) with DNA fragmentation assessed by TUNEL (r=0.83, P<0.001), percentage (r=0.77, P<0.001) and intensity of lipid peroxidation (r=0.62, P=0.009). In regard to TUNEL test, we observed a positive significant correlation between DNA fragmentation assessed by TUNEL with percentage of lipid peroxidation (r=0.84, P<0.001). In addition, there was a positive significant correlation between percentage of CMA3- positive spermatozoa with intensity of lipid peroxidation (r=0.5, P=0.03).

**Table 2:** Relationship between sperm parameters with sperm functional tests such as DFI and TUNEL+ and deficient protamine spermatozoa, persistent histones, lipid peroxidation (n=20)


Parameters	Concentration (10^6^/ml)	Total motility (%)	Abnormal morphology (%)

Persistent histones (%)	-0.56^*^	-0.50	0.54^*^
DFI (%)	-0.11	-0.45	0.37
TUNLE^+^(%)	-0.22	-0.37	0.23
CMA3^+^ (%)	-0.43	-0.28	0.47
Lipid peroxidation (%)	-0.19	-0.43	0.32
Lipid peroxidation (intensity)	-0.43	-0.45	0.62^*^


The asterisks at the end of the correlation indicate that the correlation is significant at P<0.05. DFI; DNA fragmentation index, TUNEL; Terminal deoxynucleotidyl transferase dUTP nick end labeling, and CMA3; Chromomycin A3.

**Table 3:** Correlation between sperm lipid peroxidation, DNA fragmentation and chromatin status


Parameters	Blue-stained (%)	DFI (%)	TUNEL^+^(%)	CMA3^+^(%)	Lipid peroxidation (%)

Persistent histones (%)	1	0.45	0.44	0.60^*^^*^	0.30
DFI (%)	0.45	1	0.83^*^^*^	0.30	0.77^*^^*^
TUNLE^+^(%)	0.44	0.83^*^^*^	1	0.33	0.84^*^^*^
CMA3^+^(%)	0.60^*^^*^	0.30	0.33	1	0.06
Lipid peroxidation (%)	0.30	0.77^*^^*^	0.84^*^^*^	0.6	1
Lipid peroxidation (Intensity)	0.60^*^^*^	0.62^*^^*^	0.34	0.50^*^	0.23


The asterisks at the end of the correlation indicate that the correlation is significant at * P<0.05 and **P<0.01. DFI; DNA fragmentation index, TUNEL; Terminal deoxynucleotidyl transferase dUTP nick end labeling, CMA3; Chromomycin A3, and LO; Lipid peroxidation.

## Discussion

Oxidative stress has been reported in 30-80% of infertile men and has toxic effects on sperm functions. Oxidative stress is mainly mediated through endogenous generation of hydrogen peroxide. Medium or moderate concentrations of hydrogen peroxide could result in sperm immobilization due to depletion of ATP and reduction of the phosphorylation in axonemal proteins while high concentrations of hydrogen peroxide can induce apoptosis in sperm (15). 

In the normal condition, numerous antioxidants present in seminal plasma and sperm, support male gametes against oxidative stress. However, reduced antioxidant capacity and excessive generation of ROS, prone sperm to damage in infertility condition. One of the main reasons of sperm susceptibility to damage is the low volume of cytoplasm and high content of unsaturated fatty acids (16, 17). Excessive production of ROS could also be related to mitochondrial dysfunction leading to lipidperoxidation, the consequence of which is decreased sperm motility, increased DNA fragmentation and finally apoptosis (18, 19). High level of SDF is considered as one of the main factors contributing to male infertility and can result in failed fertilization, retreaded embryonic development and consequently reduced implantation and pregnancy rates (7). Considering, traditional semen analysis is not sufficient for evaluation of sperm function and male fertility potential, we assessed sperm lipid peroxidation, protamine deficiency, and DNA fragmentation as sperm functional tests, in addition to sperm parameters, in infertile men with at least one failed cycle after ICSI.

Our results show a negative significant correlation between percentage of sperm persistent histones with sperm concentration while similar correlation was not observed between percentage of CMA3 positive spermatozoa with sperm concentration. According to literature background, aniline blue dye discriminates lysine-rich histones from arginine-and cysteine-rich protamine, while CMA3 dye compete with the protamines for binding to the minor groove of DNA in sperm (20, 21). Despite a positive significant correlation between these two markers, these results may suggest that aniline blue staining may be a better marker of sperm immaturity compared to CMA3, but one should not ignore specific group of couples with at least one or more failed cycle after ICSI and low number of cases as one limitation of this study. Our data further indicate that the chance of selection and insemination of immature sperm increases with severity of oligozoospermia. In this regard, Simon et al. (22) demonstrated that percentage of sperm persistent histones can have adverse effect on embryo development and clinical pregnancy outcomes. Unlike result of the current study, they did not observe significant correlation between sperm concentration with percentage of sperm persistent histones but they observed positive significant correlations between this parameter with DNA fragmentation assessed by three different methods (Comet, TUNEL and FCCE assays). They concluded that assessment of chromatin condensation by aniline blue staining is a good predictor of assisted reproduction technique outcomes. 

In addition, we also observed significant positive correlations between percentage of sperm abnormal morphology with percentage of sperm persistent histones and intensity of lipid peroxidation. These results suggest that abnormal sperm contain high level of excessive histones with more relaxed chromatin configuration compared to sperm chromatin that was packed with protamines, producing higher amount of hydrogen peroxide which prone sperm to lipid peroxidation. Based on previous study by professor Aitken group, lipid peroxidation by product not only exposed DNA to damage but also induces mitochondrial to produce higher amount of H_2_O_2_ , the consequence of which DNA fragmentation and apoptosis. Therefore, antioxidant therapy to minimize the level of oxidative stress has been suggested for these type of patients. In this regard, we recently demonstrated that supplementation of One-Carbone Cycle, which improves chromatin remodeling and allowse proper exchange of histone to protamine to take place resulting in the reduction of sperm lipid peroxidation and DNA damage in varicocelized rat model (23). Similar to this study, other studies showed that antioxidant therapy can improve sperm parameters, and chromatin status, and level of oxidative stress (24-28). In this study, we did not assay effect of antioxidant therapy on infertile men with previous failed cycle after ICSI. Further studies are needed to confirm this result in this group of infertile men with high population. 

According to the literature background, the final consequence of the increased level of oxidative stress and protamine deficiency is fragmentation of DNA in sperm. Therefore, we assessed SDF by two methods; TUNEL, and SCSA and observed there was a strong significant correlation between these methods. In addition, there were significant correlations between percentage of DNA fragmentation assessed by two methods with percentage and intensity of sperm lipid peroxidation. This result shows that the intensity of lipid peroxidation in sperm is in line with the fragmentation of DNA. Based on previous proposed theory by professor Aitken group, the lipid peroxidation is induced mainly by H_2_O_2_ derived from mitochondrial and leucocytes (29). Therefore, supplementation with antioxidant may break lipid peroxidation chain and subsequently may improve semen quality. Indeed, vitamin E, lycopene and astaxanthin have been recommended in the hope of protecting sperm from lipid peroxidation damage and improving fertility outcomes in these individuals (30, 31). In this regard, it has been shown that sperm with fragmented DNA could successfully complete the fertilization process, but development to reach blastocyst or post implantation is severally retarded (32, 33). A second strategy, after antioxidant supplementation, to improve ICSI outcome in these type of couples is to take the advantage of novel sperm processing methods which minimizes the load of DNA damage is selected sperm population for insemination (34). However, if these two approaches fail to result in healthy delivery, use of testicular sperm instead of ejaculated sperm (35) has been recommended. It is also important to note that changes in lifestyle which reduced the production of excessive ROS and any other action like varicocelectomy, is necessary and should be taken as the first measure in these couples (24, 36, 37).

## Conclusion

The result of these studies clearly showed that there are strong significant correlations between oxidative stress, chromatin packaging and DNA fragmentation in sperm sample of infertile men with at least one failed cycle after ICSI. It seems a reduction of oxidative stress through clinical approach like varicocelectomy and therapeutic approaches like antioxidant therapy and subsequently improvement of sperm function can be expected to provide satisfactory results in the next assisted reproduction cycle.
